# Variabilities in Retinal Hemodynamics Across the Menstrual Cycle in Healthy Women Identified Using Optical Coherence Tomography Angiography

**DOI:** 10.3390/life15010022

**Published:** 2024-12-28

**Authors:** Vlad Constantin Donica, Alexandra Lori Donica, Irina Andreea Pavel, Ciprian Danielescu, Anisia Iuliana Alexa, Camelia Margareta Bogdănici

**Affiliations:** 1 Department of Ophthalmology, Faculty of Medicine, University of Medicine and Pharmacy “Grigore T. Popa”, University Street, No. 16, 700115 Iasi, Romania; 2 Department of Rheumatology, Faculty of Medicine, University of Medicine and Pharmacy “Grigore T. Popa”, University Street, No. 16, 700115 Iasi, Romania

**Keywords:** optical coherence tomography angiography, retinal perfusion density, choroid thickness, physiological modifications, retinal microcirculation

## Abstract

**Background:** Numerous conditions, both physiological and pathological, can influence changes in the retinal vascular architecture. In order to be able to highlight pathological aspects of systemic diseases with ocular activity, it is necessary to understand how physiological fluctuations can influence circulation at the retinal level. The present study attempts to evaluate retinal and choroidal vascular and structural changes in healthy female subjects over the course of a menstrual cycle using OCT-A. **Methods:** We analyzed 22 eyes from healthy reproductive women with a regular menstrual cycle. We performed five OCT-A scans of the subjects every 7–8 days over the course of a month starting from the first day of the menstrual cycle and ending with the first day of the next cycle, measuring perfusion density in the superficial and deep vascular plexuses, choroidal thickness, and FAZ perimeter. **Results:** There are physiological variations in retinal hemodynamics that can be identified using OCT-A, choroidal thickness having statistically significant increased values in the parafoveal nasal sector during the ovulatory phase (289.18 µm) compared to the early follicular phase (281.9 µm), and the subfoveal sector during the ovulatory phase (319.04 µm) compared to the early follicular phase (308.27 µm). **Conclusions:** These findings along with abnormally small FAZ perimeters indicate that the menstrual cycle phase should be considered whenever interpreting OCT-A results. Further studies that include larger cohorts, control groups, and hormone serum levels are necessary to confirm and correlate retinal vascular alterations and the phase of the menstrual cycle using OCT-A.

## 1. Introduction

The retina has been the focus of many recent studies that have tried to evaluate structural and vascular modifications that could act as biomarkers in diagnosing and monitoring a large number of systemic diseases. In order to better recognize and understand these modifications, an increase in the current knowledge state of physiological vascular changes is required.

Many studies have proved the existence of sex hormone receptors in ocular tissues such as conjunctiva, cornea, iris, ciliary body, lens, and lacrimal glands [1,2,3]. Therefore, in the presence of hormonal imbalance, studies have shown an increased prevalence of ocular afflictions, especially dry eye disease and cataract. Circulating hormone fluctuations have been shown to have a variable effect on these ocular structures, but due to technological limitations, few studies have tried to observe these effects on the retina.

The protective properties of sex hormones over the retinal structures have been reported, normal estrogen levels being linked with a lowering in the progression of diseases such as age-related macular degeneration, macular hole, glaucoma, and diabetic retinopathy. In some cases, sex hormones can be associated with an increase in disease incidence such as the relationship between testosterone and central cerous chorioretinopathy [4].

Optical coherence tomography angiography (OCT-A) is a noninvasive imaging investigation that generates high-resolution mapping of the retinal and choroidal structure and micro-vascularization. In addition to assessing the structural and thickness abnormalities in the retinal and choroidal layers, it is able to provide information regarding vascular and perfusion density parameters in the superficial vascular plexus (SVP), deep vascular plexus (DVP), choriocapillaris plexus (CCP), and choroidal plexus (CP). These vascular structures are formed from the vascular density that irrigates their corresponding tissues [5]. OCT-A has proven useful in identifying changes in many systemic diseases with ocular modifications such as multiple sclerosis [6], autoimmune diseases [7], or cerebral vascular disorders [8]. In addition, the analysis of retinal vessel diameter using OCT-A could help in the screening of asymptomatic patients with cardiovascular disease, highlighting changes in the small retinal vessels [9]. In severe clinical depression, OCT-A modifications had a significant correlation with an increase in disease severity, especially in women over the age of 40, highlighting the protective role of sex hormones [10].

This study aims to present physiological modification found in the retinal vasculature in healthy reproductive women. The novelty of our study relies on the attempt to evaluate retinal vascular and structural changes in healthy female subjects, by analyzing these parameters weekly, five times over the course of a menstrual cycle using OCT-A.

## 2. Materials and Methods

This study was conducted from 15 January 2024 to 1 April 2024 at the “Sf. Spiridon” emergency clinical hospital. The study design and protocol were performed according to the tenets of the Declaration of Helsinki for research involving human subjects and approved by the Ethics Committee of “Grigore T. Popa” University of Medicine and Pharmacy Iasi, Romania (no. 331/12 July 2023). Subjects have signed informed consent prior to examination.

We analyzed 22 eyes from 11 healthy Caucasian reproductive women aged 25–35 years with a regular menstrual cycle (28–30 days). The inclusion criteria required a best corrected visual acuity of 20/20 and refractive errors under 4 diopters (D) spherical equivalent (SE). The exclusion criteria comprised the presence of other ocular diseases, systemic diseases such as diabetes or hypertension, and a positive medication history of oral contraceptives, hormonal therapy, or other systemic drugs. We performed 5 OCT-A scans of the subjects every 7–8 days over the course of a month starting from the first day of the menstrual cycle and ending with the first day of the next cycle. The measurements were mainly performed between morning and noon, with some cases being evaluated in the afternoon.

**Figure 1:**
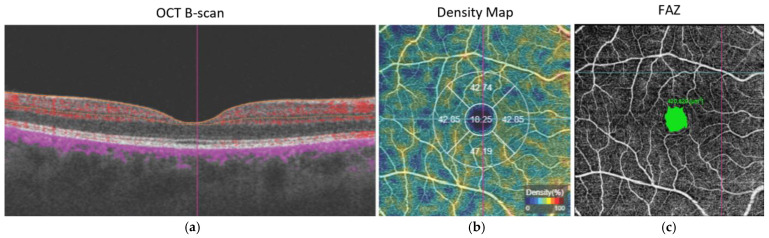
OCT-A plexus segmentation (**a**), perfusion density (**b**), and FAZ measurement (**c**).

All OCT-A scanning sessions were performed by an experienced technician using the TRITON swept source OCT device with the OCTARA software, each session having 3 scanning types. Macular cube 7 × 7 mm was used for the structural analysis of the central retina while Angiography 3 × 3 mm centered on the disc and Angiography 6 × 6 mm centered on the fovea were performed to evaluate vascular density parameters. The investigations provided information regarding retinal and choroidal central thickness, PD in the SVP, DVP, and surrounding the ONH. All scans were reviewed to ensure correct segmentation and high image quality (quality index of >60). All thickness and density segmentation and measurements were performed automatically by the proprietary software except for calculations of the foveal avascular zone (FAZ) which had been performed manually using the same software tools (Figure 1).

The Triton device software measures SVP PD from 2.6 µm under the internal limiting membrane to 15.6 µm under the IPL/INL junction, while the DVP PD is measured between 15.6 µm under the IPL/INL junction and at 70.2 µm below the IPL/INL junction.

Statistical analysis was performed using SPSS statistical package, version 26.0. All data are presented as a mean ± SD (standard deviation). A one-way repeated measures ANOVA was conducted to determine whether there was a statistically significant difference in perfusion density of the SVP, DVP, and the surrounding of the ONH, FAZ perimeter, and choroidal thickness over the course of a menstrual cycle captured in 5 scanning sessions. The data were analyzed using repeated measures analysis of variance with post hoc pairwise comparisons corrected by the Bonferroni method. A level of *p*  <  0.05 was accepted as statistically significant. Greenhouse–Geisser correction and Huynh–Feldt correction were applied to adjust the degrees of freedom.

## 3. Results

We analyzed 22 eyes from 11 healthy Caucasian female patients. While the analyzed parameters presented fluctuations between days, the only parameters found to present statistically significant modifications were the subfoveal and parafoveal nasal choroidal thickness parameters which presented lower values on Days 28–30 compared to Day 15. This would suggest that the choroid is thicker during the ovulatory phase compared to the early follicular phase.

**Figure 2:**
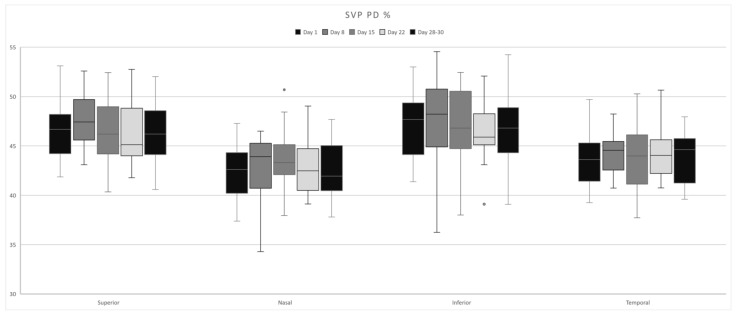
SVP–PD fluctuations in each quadrant over the course of a menstrual cycle.

SVP presented the highest PD values in the parafoveal superior, nasal, and inferior quadrants on Day 8 (Figure 2), corresponding to the follicular phase of the menstrual cycle, and the parafoveal temporal and foveal on Days 28–30, corresponding to the early follicular phase (Table 1). No statistically significant modifications were found.

**Figure 3:**
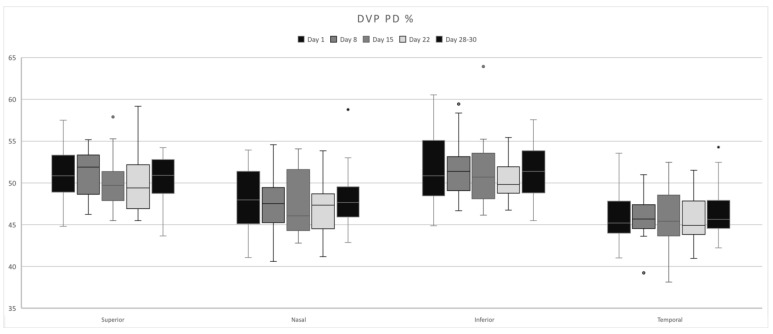
DVP–PD fluctuations in each quadrant over the course of a menstrual cycle.

**Figure 4:**
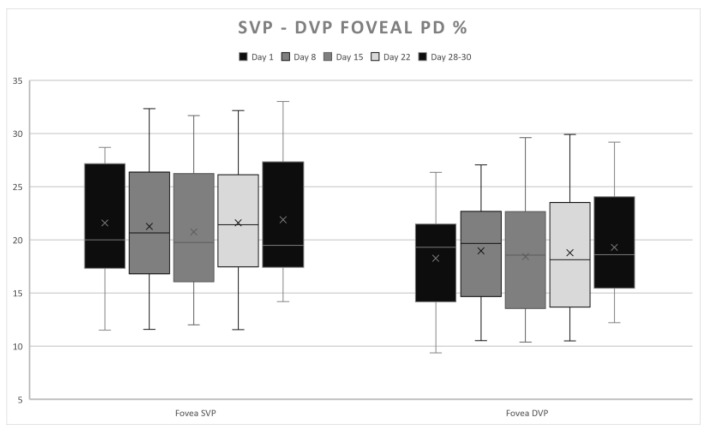
SVP and DVP–PD fluctuations in the fovea over the course of a menstrual cycle.

DVP presented the highest PD values in the parafoveal superior quadrant on Day 8 (Figure 3), the nasal and inferior quadrants on Day 1, and the parafoveal temporal and foveal (Figure 4) on Days 28–30, corresponding to the early follicular phase (Table 2). No statistically significant modifications were found.

**Figure 5:**
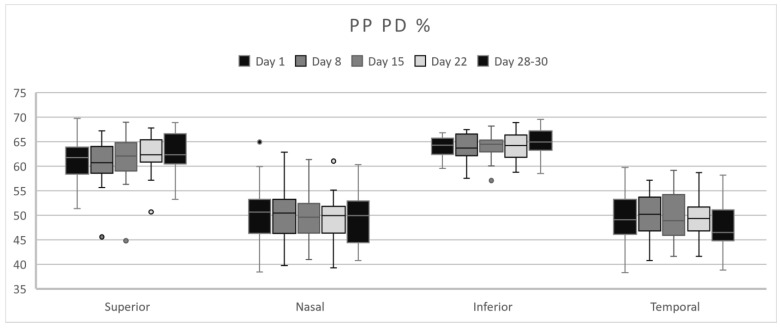
PP–PD fluctuations in each quadrant over the course of a menstrual cycle.

The PP area presented the highest PD values in the parafoveal superior and inferior quadrant on Days 28–30 (Figure 5) and in nasal and inferior quadrants on Day 8 (Table 3). No statistically significant modifications were found.

**Figure 6:**
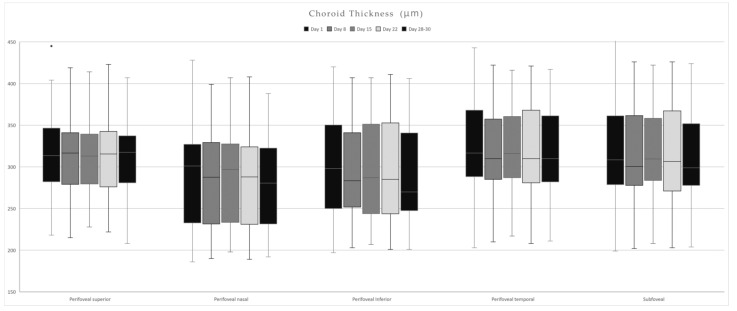
Choroidal thickness values in each quadrant over the course of a menstrual cycle.

Choroidal thickness had the highest mean values on Day 1 in all quadrants but presented no statistically significant difference when compared to other days (Table 4). Day 15 presented a statistically significant increase in thickness compared to Days 28–30 in the parafoveal nasal (*p* = 0.048) and subfoveal areas (*p* = 0.018) (Figure 6).

**Figure 7:**
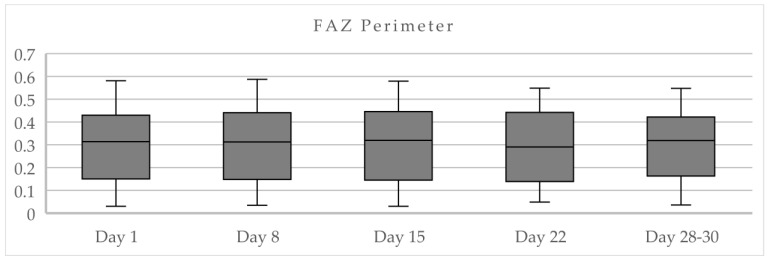
FAZ values in each quadrant over the course of a menstrual cycle.

**Figure 8:**
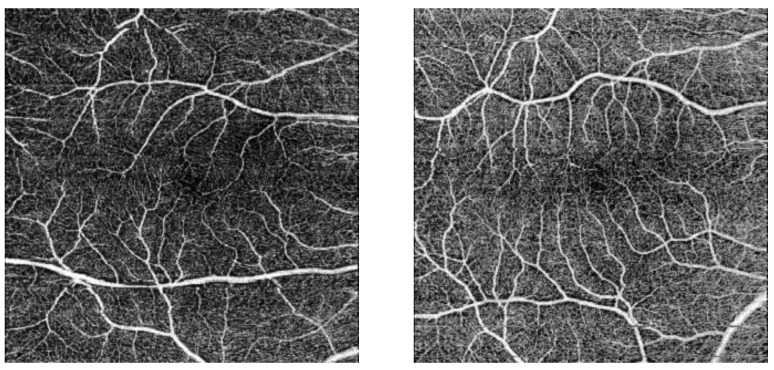
Small FAZ perimeters in healthy patients.

The FAZ perimeter mean values were between 0.284 and 0.295 over the course of a menstrual cycle (Table 5). The highest mean values correspond to the first day of the menstrual cycle (Figure 7). Four eyes presented abnormally small FAZ areas (Figure 8).

## 4. Discussion

The quantification of the values obtained by OCT is performed by comparing the values obtained with a reference interval of the healthy population depending on age, sex, and ethnicity. The values obtained using OCT-A do not have the same degree of repeatability, being influenced by various factors, both physiological and pathological. Thus, the majority of studies have focused on serial imaging capturing changes occurring over time.

In the female population, changes in the retinal circulation were identified depending on the activity of sex hormones. Within the menstrual cycle, Guo et al. identified a lower perfusion density in DVP during the ovulatory phase compared to the luteal phase in the inferior and nasal quadrants of the macular area. These changes would be secondary to hormonal fluctuations and are considered physiological [11]. Kurahashi et al. identified a decrease in choroidal thickness during the luteal phase of the menstrual cycle [12]. A significantly decreased choroidal thickness was found by Ulaş et al. when comparing the mid-luteal phase to the early follicular and ovulatory phases [13]. Our study was unable to support these previous findings, due to not having any statistically significant results regarding DVP or choroidal thickness in the luteal phase compared to other stages. This aspect could be caused by a difference in OCT measurement and segmentation, with our study having an OCT based on swept-source technology, while the other three studies measured using a spectral domain machine. While the number of analyzed eyes in the study by Guo et al. was 62, Kurahashi et al. and Ulaş et al. presented a comparable number of analyzed cases. Therefore, the low number of participants in these studies may be another factor for a difference in results.

Haneda et al. used laser speckle flowgraphy to assess choroidal blood flow velocity and found an increase in the luteal phase compared to the late follicular phase [14].

In postmenopausal women, an improvement in retinal circulation has been observed following estrogen hormone therapy compared to individuals without therapy. They show increased RNFL thickness, with increased vascular perfusion around the inferior temporal retinal artery branch [15]. Fortepiani et al. compared female subjects on contraceptives with subjects with normal menstrual cycles. The contraceptive group showed reduced foveal thickness compared to the control group [16]. These results were also found by Shaaban and Badran, observing a reduction in the thickness of all retinal parameters, especially those in the macular area [17]. During the last trimester of pregnancy, a decrease in PD from SVP was observed, with a concomitant increase in PD from DVP [18]. In analyzing patients with polycystic ovary disease, Yener et al. did not identify vascular abnormalities but found significant parafoveal thickening in all but the nasal quadrants [19].

Considering the stages of menstruation or hormonal levels in female patients during the interpretation of OCT-A results could justify certain inconsistencies, reducing the number of errors, thus emphasizing the importance of estrogen hormones in retinal homeostasis. Through OCT-A, Baker et al. identified changes in retinal vascular plexuses due to hypoxia installed at high altitudes. They objectified an increase in retinal circulation density in people who spent more than two days at altitudes above 3800 m. Thus, the changes found appeared late compared to hypoxia in the general circulation but were maintained over time [20]. The increase in retinal perfusion was also objectified by Xie et al., who highlighted a correlation between the increase in peripapillary vascular perfusion and the severity of acute altitude sickness, which could be a sign of cerebral edema [21].

Bujor et al. performed a comparative analysis of the retinal microvasculature between healthy Chinese and Caucasian subjects using OCT-A. They identified significant microvascular differences, especially in the SVP and FAZ. While our study only contained Caucasian female patients, the consideration of ethnicity along with age and gender in future studies is essential for the correct interpretation of OCT-A results [22].

OCT-A has also been used to establish the early stabilization of operative success in patients with compressive pituitary adenoma. Cennamo et al. have objectified an increase in the VD of the retinal plexuses that occurs early after surgery compared to retinal structural changes objectified by OCT [23].

The large number of exploratory options using OCT-A has caused a multitude of new parameters to appear, thus being difficult to analyze. Future directions should focus on automated programs and machine learning techniques that would facilitate the recognition of new biomarkers in different pathologies with systemic affliction that determine changes at the retinal level [24]. The main benefits of using such programs lie in their ability to continuously optimize the obtained results by adding new values and parameters. Arian et al. highlighted these properties using information captured by OCT and infrared laser ophthalmoscopy scanning images observing an increase in automated retinal lesion recognition systems in multiple sclerosis [25].

The limits of this study consist in the low number of participants, the lack of testing for the proper correlation of menstrual cycle stages and OCT-A captures, and the lack of a control group. In addition, the manual measurement of the FAZ perimeter may represent another source of error. While all subjects did present a 28–30-day menstrual cycle during our study, this had only been registered based on patient statements and measuring serum hormone levels would be required to corelate the retinal vascular modifications with hormonal activity. In addition, although the results do not differ in statistical significance, there are small differences between the first day of the menstrual cycle and the first day of the next cycle (Day 1 vs. Days 28–30); therefore the study would require a larger period of monitoring for the repeatability of the vascular fluctuations.

Future directions should focus on including a larger study population and control groups that have a similar timetable for OCT-A captures, where sex hormone levels remain unchanged, such as male population or menopausal women. Additionally, there should be a proper correlation between hormone levels and capture dates in order to establish menstrual cycle stages. Moreover, focusing on concurrent factors like age, number of pregnancies, or baseline vascular parameters could lead to the discovery of correlations between such parameters.

Through OCT-A, we tried to highlight changes in the retinal circulation that are independent from other systemic pathology activity. Numerous conditions, both physiological and pathological, can influence microvascular changes in the retinal plexuses. In order to highlight the pathological aspects of the inflammatory episodes, it is necessary to understand how these fluctuations can influence circulation at the retinal level.

## 5. Conclusions

Our study suggests that there are physiological variations in retinal hemodynamics in healthy reproductive females that can be identified using OCT-A, with choroidal thickness having statistically significant increased values in the ovulatory phase compared to the early follicular phase in the parafoveal nasal and subfoveal areas. These findings, along with abnormally small FAZ perimeters, indicate that the phase of the menstrual cycle should be considered when interpreting OCT-A results. Further studies that include larger cohorts, control groups, and hormone serum levels are necessary to confirm and correlate retinal vascular alterations and the phase of the menstrual cycle using OCT-A.

## Figures and Tables

**Table 1:** SVP–PD mean values ± SD over the course of a menstrual cycle.

SVP %	Day 1		Day 8		Day 15		Day 22		Days 28–30	
	Mean	SD	Mean	SD	Mean	SD	Mean	SD	Mean	SD
Parafoveal Superior	46.51	2.71	**47.42**	2.77	46.4	3.14	46.42	3.04	46.10	2.89
Parafoveal Nasal	42.33	2.69	**42.74**	3.30	43.65	2.86	42.71	2.58	42.54	2.93
Parafoveal Inferior	46.91	3.23	**47.58**	4.24	47.00	3.80	46.43	2.84	46.41	3.75
Parafoveal Temporal	43.66	2.74	44.1	2.00	44.09	3.21	44.20	2.38	**43.87**	2.55
Foveal	21.58	5.30	21.26	5.97	20.74	5.60	21.59	5.87	**21.89**	5.89

**Table 2:** DVP–PD mean values ± SD over the course of a menstrual cycle.

DVP %	Day 1		Day 8		Day 15		Day 22		Days 28–30	
	Mean	SD	Mean	SD	Mean	SD	Mean	SD	Mean	SD
Parafoveal Superior	51.12	3.21	**51.33**	2.74	50.05	3.01	50.05	3.74	50.61	2.71
Parafoveal Nasal	**47.99**	3.53	47.41	3.52	47.65	3.88	46.81	2.8	47.98	3.52
Parafoveal Inferior	**51.59**	4.34	51.54	3.38	51.43	3.9	50.33	2.23	51.45	3.56
Parafoveal Temporal	45.98	3.16	45.93	2.51	45.67	3.57	45.65	2.91	**46.37**	3.08
Foveal	18.26	4.86	18.97	4.83	18.43	5.4	18.79	5.31	**19.29**	4.94

**Table 3:** Peripapillary PD mean values ± SD over the course of a menstrual cycle.

PP %	Day 1		Day 8		Day 15		Day 22		Days 28–30	
	Mean	SD	Mean	SD	Mean	SD	Mean	SD	Mean	SD
Superior	61.10	4.42	60.49	4.61	61.30	4.86	62.31	3.83	**62.55**	4.06
Nasal	50.09	6.15	**50.25**	5.96	49.61	4.78	49.23	4.93	49.19	5.15
Inferior	64.02	2.10	63.90	2.58	63.84	2.50	64.12	2.59	**65.15**	2.84
Temporal	49.05	5.18	**49.82**	4.48	49.54	4.84	49.61	4.01	47.82	4.97

**Table 4:** Choroid thickness mean values ± SD over the course of a menstrual cycle.

Choroid Thickness	Day 1		Day 8		Day 15		Day 22		Days 28–30	
µm	Mean	SD	Mean	SD	Mean	SD	Mean	SD	Mean	SD
Parafoveal Superior	**316.81**	51.48	313.36	46.55	305.59	48.20	310.95	48.39	309.5	47.56
Parafoveal Nasal	**290.68**	65.64	286.22	60.31	289.18	61.79	286.09	60.88	281.90	58.55
Parafoveal Inferior	**300.27**	62.23	296.81	61.79	298.18	63.33	297.95	65.78	293.22	62.92
Parafoveal Temporal	**322.50**	60.54	316.90	55.70	317.81	56.40	315.71	57.49	311.13	54.62
Subfoveal	**319.04**	66.00	313.22	59.63	**319.04**	60.79	315.09	61.36	308.27	56.12

**Table 5:** FAZ perimeter mean values ± SD over the course of a menstrual cycle.

	Day 1		Day 8		Day 15		Day 22		Days 28–30	
FAZ mm^2^	Mean	SD	Mean	SD	Mean	SD	Mean	SD	Mean	SD
	**0.295**	0.173	0.286	0.171	0.289	0.175	0.284	0.165	0.285	0.158

## Data Availability

The data published in this research are available on request from the first author.
